# Chronic catatonia treated with electroconvulsive therapy: a case report

**DOI:** 10.1186/1752-1947-7-219

**Published:** 2013-08-23

**Authors:** Varuni A de Silva, Wickramaarachchige D Lakmini, Heshan N Gunawardena, Raveen Hanwella

**Affiliations:** 1 Department of Psychological Medicine, Faculty of Medicine, University of Colombo, Kynsey Road, Colombo 08, Sri Lanka; 2 University Psychiatry Unit, National Hospital of Sri Lanka, Hospital Square, Colombo 10, Sri Lanka

**Keywords:** Chronic catatonia, Electroconvulsive therapy, Schizophrenia

## Abstract

**Introduction:**

In the International Statistical Classification of Diseases and Related Health Problems 10 and *Diagnostic and Statistical Manual of Mental Disorders* IV classification systems, catatonia is classified as a subtype of schizophrenia. However, catatonia is more frequently associated with mood disorders than schizophrenia. It is also associated with organic conditions. Catatonia responds to treatment with benzodiazepines and electroconvulsive therapy rather than antipsychotics. These features support the categorization of catatonia as an independent syndrome. There is a lack of consensus regarding the definition of chronic catatonia. There are two previous case reports of effective treatment of chronic catatonia with electroconvulsive therapy.

**Case presentation:**

A 29-year-old South Asian woman was admitted to hospital because of poor food intake. Her condition had progressively worsened over the past seven months. She had features of catatonia. On admission, her Bush-Francis Catatonia Rating Scale score was 24. Her symptoms resolved after the administration of 17 electroconvulsive therapies but recurred later. She was given a further four electroconvulsive therapies. She remains well on aripiprazole at a dose of 60mg a day.

**Conclusions:**

Bilateral electroconvulsive therapy is effective in the treatment of chronic catatonia and should be considered as a treatment option. A relapse of symptoms can occur after the discontinuation of treatment.

## Introduction

*The Diagnostic and Statistical Manual of Mental Disorders, fourth edition* (DSM IV) describes catatonia as a syndrome with marked psychomotor disturbance that may involve motor immobility, excessive motor activity, extreme negativism, mutism, peculiarities of voluntary movement, echolalia or echopraxia [1].

Catatonia was first described by Kahlbaum as a disorder of movement and speech. Kraepelin grouped together catatonia, hebephrenia and paranoid psychosis, as dementia praecox. Eugen Bleuler later coined the term schizophrenia for this condition.

In the International Statistical Classification of Diseases and Related Health Problems (ICD-10) and DSM IV classification systems, catatonia is classified as a subtype of schizophrenia [1,2]. However catatonia is more frequently associated with mood disorders than schizophrenia [3]. It is also associated with organic conditions. Unlike other subtypes, the core features of schizophrenia, delusions and hallucinations, are not characteristic of catatonia. Catatonia responds to treatment with benzodiazepines and electroconvulsive therapy (ECT) rather than antipsychotics. These features support the categorization of catatonia as an independent syndrome [3].

Taking into account the current scientific evidence, the DSM-5 made several changes in the classification of catatonia [4]. ‘Schizophrenia, catatonic type’ has been eliminated. Catatonia is included as a specifier across the 10 principal primary diagnoses. The subcategory ‘catatonia associated with another mental disorder’ is retained and a new subcategory of ‘unspecified catatonia’ has been included.

Benzodiazepines are effective in the treatment of acute catatonia [5]. The motor symptoms of acute catatonia probably occur due to a deficiency of cortical gamma-aminobutyric acid (GABA). Benzodiazepines increase GABA activity, which could explain their therapeutic effect in catatonia. Some patients with chronic catatonia require higher doses of lorazepam administered for long durations [6-8]. Manjunatha *et al*. report that a 28-year-old woman with recurrent catatonia responded initially to lorazepam 4mg a day, but symptoms recurred when the dose was reduced to 2mg a day [7]. The catatonic episodes recurred five times in six months and each time the symptoms responded to lorazepam 6mg a day. Mukai *et al*. reported that a patient with chronic catatonia responded to daily doses of clozapine 300mg and lorazepam 1mg [8]. Grover *et al*. report that a 38-year-old woman with chronic catatonia responded to lorazepam 6mg a day, imipramine 175mg a day and risperidone 4mg a day. This patient developed catatonic symptoms each time the lorazepam was tapered off, therefore she was maintained on lorazepam for more than two years [6]. Another case report describes that lorazepam was effective in the treatment of chronic ‘speech catatonia’ [9]. Gaind *et al*. report that chronic catatonia in a mentally retarded man responded to high doses of lorazepam after five months of treatment [10]. However, a 12-week crossover randomized trial found that lorazepam 6mg a day was not effective in the treatment of chronic catatonia [4].

There are only two previous case reports of chronic catatonia treated with ECT. One patient was a 41-year-old Asian woman with schizophrenia with intermittent catatonic symptoms of 12 years’ duration [11]. She was treated successfully with eight ultrabrief pulse width right unilateral treatments given at six times seizure threshold. The other case report is of a 25-year-old woman with features of catatonia who was administered bilateral ECT [12]. In this patient, bilateral ECT was administered twice a week (impulse width 0.5 msec, energy set first 53 treatments 5% to 100% and second 15 treatments 5% to 35%). The catatonic symptoms reappeared when ECT was stopped, therefore ECT was restarted along with olanzapine 10mg a day. Other than these case reports, there are no clinical trials regarding the use of ECT in chronic catatonia.

Since there are only a few reports of chronic catatonia and its treatment, we describe the successful treatment of a patient with catatonic symptoms of over six months with ECT.

## Case presentation

A 29-year-old South Asian woman was admitted to hospital because of poor food intake. Her condition had progressively worsened over the past seven months. The family reported that she was very slow in her movements. She kept food in her mouth without chewing or swallowing. She also maintained the same posture for long periods. Her speech had gradually reduced in quantity. In the month prior to admission, she was unable to carry out activities such as eating, bathing and dressing without help. There was no history of fever, loss of consciousness or seizures.

On examination, the patient was mute and staring vacantly into space. Her movements were very slow. She maintained uncomfortable postures for long periods, such as the psychological pillow and holding her arms over her head in a prayer-like manner. There was automatic obedience and mitgehen. Tone was increased in all four limbs and waxy flexibility was present. There was no echolalia, echopraxia, mannerisms or stereotypy. There were no periods of motor excitement. There were no focal neurological signs. Her cardiovascular, respiratory system and abdominal examination were normal. On admission, her Bush-Francis Catatonia Rating Scale score was 24.

A full blood count, liver function, renal function, fasting blood sugar, erythrocyte sedimentation rate (ESR) and a computed tomography (CT) scan of the brain were all normal. Antinuclear antibody tests were negative. Her short Synacthen test and thyroid function test (TSH, T3, T4) results were normal.

She first developed psychotic symptoms in 2008. Treatment was commenced in 2009 but, after four months, she defaulted on treatment. She became pregnant in 2010. Although she had paranoid symptoms during the antenatal period, she did not seek treatment. Her symptoms worsened after the delivery of the baby. Her movements became slow, she was withdrawn and her self-care was poor. Because of the illness, she was unable to care for the baby. In June 2011, seven months after the delivery of her baby, she was admitted to hospital with catatonic features. She recovered after administration of four ECTs. She defaulted on treatment again in November 2011 and became paranoid and withdrawn. Her level of functioning gradually deteriorated.

By April 2012, she was almost mute and needed help to carry out activities such as eating and dressing. The family sought psychiatric treatment and she was treated with mirtazapine 15mg for three weeks and then venlafaxine 150mg and lithium carbonate 400mg for three months. Since there was poor response, treatment was changed to risperidone. Because her condition deteriorated further, she was admitted to hospital in November 2012.

After admission to hospital, the patient was treated with oral lorazepam 4mg twice a day. There was only slight improvement in speech and intake of food after seven days of treatment. ECT was then commenced using a Thymatron System IV machine (Somatics Inc., Lake Bluff, IL, USA). Bilateral ECT was administered three times a week. Initially, 15% energy was given (76mC) and this was gradually increased to 200% (1008mC) because she did not have adequate seizures. After 10 ECTs, significant reduction in catatonic symptoms was noted, the patient was able to feed herself, communicate with others and her self-care improved. Her Bush-Francis Catatonia Rating Scale score was 12. She received a total of 17 ECTs. By this time, most of the catatonic symptoms had disappeared and her Bush-Francis Catatonia Rating Scale score was 3. Olanzapine was commenced and gradually increased to 30mg a day. She was discharged after one month in hospital.

Two weeks after discharge olanzapine was stopped because she developed galactorrhoea. Aripiprazole was commenced and the dose was gradually increased to 60mg a day because of the reappearance of slowness, reduced speech and rigidity. Since the symptoms did not improve, she was readmitted and a further four ECTs were given, after which the catatonic symptoms disappeared. She continues to be well on aripiprazole 60mg a day.

## Discussion

This patient, with catatonic symptoms of six months duration, showed poor response to antidepressants and antipsychotics. Treatment with lorazepam for seven days did not result in any significant improvement. She recovered after the administration of 17 ECTs.

Since the DSM −5 does not contain a category of ‘schizophrenia, catatonic type’, the clinical features in this patient support a diagnosis of ‘catatonia associated with another mental disorder’ using the specifier as a fifth digit [13]. Interestingly, since there is no separate category for catatonia of prolonged duration, this patient could also be classified as ‘unspecified catatonia’.

There is a lack of consensus regarding the definition of chronic catatonia. Most of the evidence about chronic catatonia is based on case reports. The duration of symptoms in these case reports vary from one to six months. Therefore, we suggest that chronic catatonia should be defined as the presence of catatonia for a minimum of 30 days.

Some patients with chronic catatonia experience continuous symptoms while others have recurrent episodes, especially when treatment is discontinued [6,7]. In our patient, the symptoms recurred about a month after the discontinuation of ECT. There is genetic evidence that MLC1 polymorphisms are specifically associated with periodic catatonia, a variety of chronic catatonia [14]. In a patient with recurrent episodes of catatonia lasting two to three months, a magnetic resonance imaging scan revealed mega cisterna magna that was mildly and diffusely compressing the left cerebellum and causing pressure scalloping of the internal table of the skull vault in the left occipital region [15]. These findings suggest that future studies should explore the concept of recurrent catatonia.

Although benzodiazepines and ECT are recommended for the treatment of acute catatonia, treatment options for chronic catatonia have not been discussed extensively. Antipsychotics do not appear to be effective in terminating chronic catatonic symptoms although they may be effective in preventing recurrence [6].

Case reports indicate that both bilateral ECT and ultrabrief pulse ECT are effective in the treatment of chronic catatonia. ECT is also effective in patients who do not have an adequate response to lorazepam [11]. Because there are only three case reports of the use of ECT in chronic catatonia, it is difficult to estimate the number of treatments needed for clinical response. One patient with chronic catatonia improved with eight ECTs while the other required 68 ECTs [11,12]. Our patient required a total of 21 ECTs. These reports suggest that some patients with chronic catatonia need a large number of ECTs for adequate clinical response.

Although the only randomized trial showed that lorazepam is not effective in the treatment of chronic catatonia, case reports indicate that, in some patients, lorazepam is effective. As with ECT, some may need to be treated for long periods [6-8,10].

## Conclusions

Bilateral ECT is effective in the treatment of chronic catatonia and should be considered as a treatment option in chronic catatonia. A relapse of symptoms can occur after the discontinuation of treatment.

## Consent

Written informed consent was obtained from the patient for publication of this case report. A copy of the written consent is available for review by the Editor-in-Chief of this journal.

## Competing interests

The authors declare that they have no competing interests.

## Authors’ contributions

WDL and HNG managed the patient and wrote the preliminary draft of the manuscript. RH and VAS were the major contributors in writing the manuscript. All authors read and approved the final manuscript.
